# Water-Based Exercises on Peak Oxygen Consumption, Exercise Time, and Muscle Strength in Patients with Coronary Artery Disease: A Systematic Review with Meta-Analysis

**DOI:** 10.1155/2023/4305474

**Published:** 2023-06-26

**Authors:** Alana Lalucha Andrade Guimarães, Mansueto Gomes-Neto, Lino Sérgio Rocha Conceição, Micheli Bernardone Saquetto, Caroline Oliveira Gois, Vitor Oliveira Carvalho

**Affiliations:** ^1^The GrEAt Group (Grupo de Estudos em Atividade Física), Brazil; ^2^Physical Therapy Department, Federal University of Sergipe, Brazil; ^3^Post-Graduate Program in Health Sciences, Federal University of Sergipe, Brazil; ^4^Physical Therapy Department, Federal University of Bahia, Brazil

## Abstract

**Background:**

There is a growing use of water-based exercises in cardiac rehabilitation programs. However, there is little data concerning the effects of water-based exercise on the exercise capacity of coronary artery disease (CAD) patients.

**Objective:**

To perform a systematic review to investigate the effects of water-based exercise on peak oxygen consumption, exercise time, and muscle strength in patients with CAD.

**Methods:**

Five databases were searched to find randomized controlled trials that evaluated the effects of water-based exercise for coronary artery disease patients. Mean differences (MD) and 95% confidence intervals (CIs) were calculated, and heterogeneity was assessed using the *I*^2^ test.

**Results:**

Eight studies were included. Water-based exercise resulted in an improvement in peak VO_2_ of 3.4 mL/kg/min (95% CI, 2.3 to 4.5; *I*^2^ = 0%; 5 studies, *N* = 167), exercise time of 0.6 (95% CI, 0.1 to 1.1; *I*^2^ = 0%; 3 studies, *N* = 69), and total body strength of 32.2 kg (95% CI, 23.9 to 40.7; *I*^2^ = 3%; 3 studies, *N* = 69) when compared to no exercising controls. Water-based exercise resulted in an improvement in peak VO_2_ of 3.1 mL/kg/min (95% CI, 1.4 to 4.7; *I*^2^ = 13%; 2 studies, *N* = 74), when compared to the plus land exercise group. No significant difference in peak VO_2_ was found for participants in the water-based exercise plus land exercise group compared with the land exercise group.

**Conclusions:**

Water-based exercise may improve exercise capacity and should be considered as an alternative method in the rehabilitation of patients with CAD.

## 1. Background

Coronary artery disease represents an important cause of death and disability worldwide. Besides current medical intervention, lifestyle change plays a key role in the prevention and rehabilitation of this condition, such as smoking cessation, dietary interventions, and physical activity [1].

It is known that both aerobic exercise capacity and muscle strength are frequently decreased and represent important prognostic variables in patients with coronary artery disease [2]. The American Heart Association considers cardiorespiratory fitness as a vital sign and encourages its assessment [3]. In addition, they show the importance of prioritizing functional capacity, such as aerobic and strength capacities, as the principal end point for older adults with cardiovascular disease [4]. Thus, exercise-based cardiac rehabilitation for patients with coronary artery disease is an effective low-cost intervention that can reduce cardiovascular mortality and the risk of hospital admission [5]. Classically, land-based aerobic and strength exercise training are the most prescribed method of rehabilitation. However, less frequently prescribed modalities, such as water-based exercises, are growing up in importance on the scientific literature [6–8].

Water-based exercise is a safe and efficient modality of exercise intervention in patients with cardiac conditions [8, 9]. The belief that immersing these patients in water would provoke cardiovascular overload is not accepted anymore [10]. Studies have been shown that immersion in water decreases afterload and improves cardiac performance [11]. Moreover, a recent systematic review showed that exercising the patient with heart failure in a water environment is effective in improving exercise capacity and quality of life [12]. Recently, Cugusi et al. [13] published a systematic review on supervised water-based exercise for men with coronary artery disease. They concluded that based on the available evidence, water-based exercise improves exercise tolerance in men with coronary artery disease. However, new trials have been published since then [14, 15]. Additionally, this study expands on previous publication increasing the accuracy of the results found by performing a comprehensive systematic literature review with meta-analysis of randomized clinical trials to investigate the effects of water-based exercises on diverse clinical outcomes. In addition, the Cochrane Handbook recommends the systematic review update within 2 years [16]. More specifically, our systematic review investigated the effects of water-based exercises plus land-based exercise and water-based exercise alone on peak oxygen consumption (peak VO_2_), exercise time, and muscle strength in patients with coronary artery disease.

## 2. Methods

This study was designed and performed in accordance with the Cochrane Handbook recommendations [16] and completed in accordance with the PRISMA (preferred reporting items for systematic reviews and meta-analysis) statement [17]. The systematic review protocol has been registered with the PROSPERO International prospective register of systematic review database (CRD42022324397).

### 2.1. Eligibility Criteria

#### 2.1.1. Population

To be eligible, the trial had to include individuals with diagnosed coronary artery disease (history of coronary artery disease with angina pectoris or myocardial infarction diagnosed by American Heart Association standard criteria [18] angiographically documented, and/or percutaneous coronary intervention).

#### 2.1.2. Intervention

We included studies with any kind of water-based exercises. We considered water-based exercises as any exercise training program performed in a water environment without temperature restrictions.

#### 2.1.3. Control Group

For the control group, studies of any kind of land-based exercises (active controls) or no intervention (usual daily activities) were included.

#### 2.1.4. Outcomes

The main outcome was exercise capacity measured by peak VO_2_ (mL/kg/min). Secondary outcomes were exercise time (minutes) by duration of the exercise test and any method of total muscle strength.

### 2.2. Study Design

We included randomized controlled trials and nonrandomized clinical trials.

### 2.3. Search Strategy and Study Selection

We searched for references on MEDLINE/PubMed, EMBASE, PEDro database, LILACS, and the Cochrane Central Register of Controlled Trials up to April 2022 without language restrictions. The strategy developed by Higgins et al. [16] was used for the identification of the trials in MEDLINE/PubMed and Cochrane. To identify the trials in EMBASE, a search strategy using similar terms was adopted. A search strategy using similar terms was also used to identify studies in other databases. A standard protocol for this search was developed, and whenever possible, controlled vocabulary (MeSH term for MEDLINE/PubMed and Cochrane and Emtree term for EMBASE) was used to build our search strategy. We used the Boolean operators “And”/“or” in combination with specific descriptors (water-based exercise, aquatic therapy, or hydrotherapy) and coronary artery disease (supplementary content (available here).

The selection of studies was performed using the Rayyan [19] selection platform. Two independent investigators (A.L.A.G. and M.G.N.) searched for eligible studies according to title and abstract. After the initial selection, the same investigators analyzed the full text to include or not the trials. Disagreements were resolved by consensus. If not possible, a third investigator (L.S.R.C.) made the decision.

### 2.4. Data Extraction

A standardized form for data extraction [16] and storage was developed using Microsoft Excel software (version 2016). For results presented in graphs, we used the WebPlotDigitizer program for data extraction [20]. Two independent investigators performed the data extraction of the included studies. Disagreements were resolved by a third reviewer. In case of missing data, the authors were contacted by email with a deadline of 14 days for a response.

### 2.5. Quality of Meta-Analysis Evidence

The quality of studies included in this systematic review was scored by two researchers using the PEDro scale, which is based on important criteria, such as concealed allocation, intention-to-treat analysis, and the adequacy of follow-up. These characteristics make the PEDro scale a useful tool for assessing the quality of rehabilitation trials [21–23]. Any disagreements in the rating of the studies were resolved by a third reviewer.

### 2.6. Statistical Assessment

Pooled-effect estimates were obtained by comparing the least square mean change from baseline to end point for each group and were expressed as the weighted mean difference between groups. When the standard deviation (SD) of change was not available, the SD of the baseline measure was used for the meta-analysis. Data were imputed when data were not available.

Calculations were done using a fixed-effect and random-effect model. If the trial was a multiple-arm randomized controlled trial, all relevant experimental intervention groups (water-based exercise plus land-based exercise or water-based exercise versus land exercise or no intervention) had data extracted. In follow-up reports with multiple end points, only data closest to the end of the exercise program were included. In cross-over trials, size effects were only extracted at the first cross-over point.

We compared water-based exercise plus land-based exercise versus land-based exercise, water-based exercise versus land-based exercise group, and water-based exercise versus no exercise (control group). An *α* value ≤0.05 was considered significant. Heterogeneity among studies was examined with Cochran's *Q* and *I*^2^ statistics, in which values greater than 40% were considered indicative of high heterogeneity [24], and random-effects model was chosen. Analyses were performed with Review Manager (version 5.4) [25].

The quality of evidence for the outcomes in meta-analysis was assessed using the Grading of Recommendations Assessment, Development and Evaluation (GRADE) approach to interpret result findings and using GRADEpro GDT 2015 to import data from the Review Manager to create a “summary of findings table” [18]. The assessment involved five items: risk of bias, imprecision, inconsistency, indirectness, and publication bias. The quality of evidence was downgraded by one level for risk of bias when more than a quarter of the studies included in the meta-analysis were considered at high risk of bias (studies without allocation concealment, random allocation, and/or sample size calculation). Results were considered imprecise if the pooled sample size was <300 for dichotomous outcomes or <400 for continuous outcomes, and inconsistent if the heterogeneity between trials was substantial (i.e., *I*^2^ > 40%). Whenever possible, publication bias was assessed by visual inspection of funnel plots (scatterplot of the ES from individual studies against its SE) for the meta-analysis with 10 or more trials [16, 26, 27]. Decisions to downgrade the quality of studies were justified using footnotes and making comments, where necessary, to aid readers' understanding of the review.

## 3. Results

### 3.1. Study Selection

**Figure 1:**
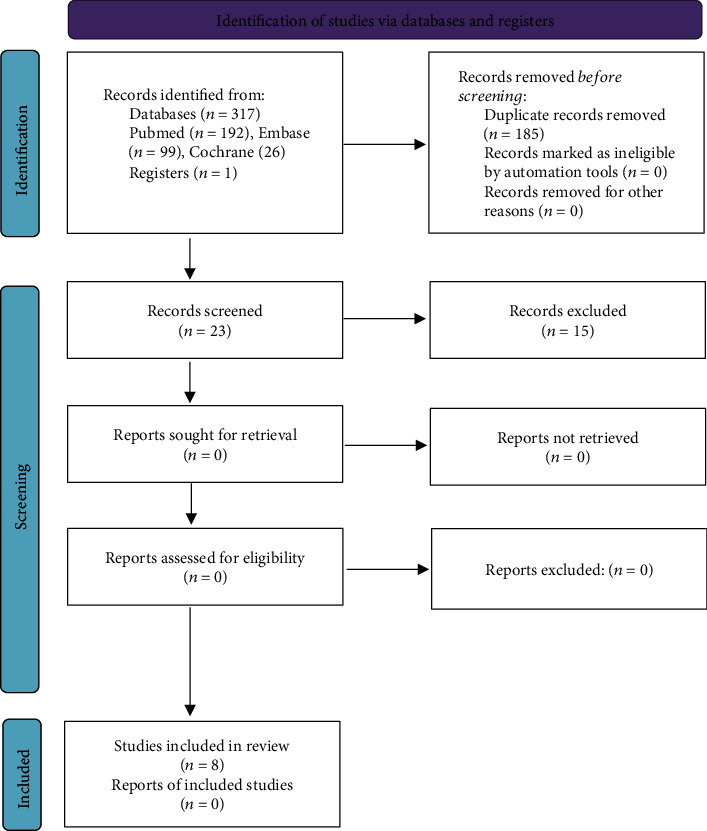
Flow diagram showing the reference screening and study selection (which included searches of databases and registers only).

The initial search led to the identification of 317 studies; after screening for duplicates, we identify 23 potentially eligible studies and 1 additional study identified by other sources. 11 studies were considered as potentially relevant after title and abstract screening and were retrieved for detailed analysis. After a complete reading of 11 articles, 3 were excluded and 7 randomized controlled trials [6, 14, 15, 28–31] and 1 nonrandomized clinical trial [32] met the eligibility criteria.  Figure 1 shows the PRISMA flow diagram of the studies included in the review. Data from included studies were then extracted. Both reviewers scored each article using the PEDro scale. The results of their assessments are presented in Table 1.

### 3.2. Study Characteristics

Eight studies were included in the review. Seven trials were randomized controlled trials [6, 14, 15, 28–31] and 1 nonrandomized clinical trial [32]. The number of participants randomized in this systematic review ranged from 21 [30, 32] to 89 [14], totaling 340 participants. All studies analyzed in this review included patients with coronary artery disease, whose ages ranged from 51.6 to 72.8 years. Five studies [6, 29–32] included only male participants. The sample sizes, outcomes, and results of the studies are summarized in Table 2.

The intervention performed was water-based exercise and was described as swimming pool and aerobic and resistance training, with a temperature of 28–34.5°C. All intervention protocols were applied under the supervision of the investigator, as shown in Table 3.

The clinical trials showed moderate methodological quality. The PEDro scale (score out of 10) showed a mean score of 4.75 (4–5), of which, 75% of studies had a score that indicated moderate methodological quality (Table 1). Most included studies showed methodological limitations such as concealed allocation, blinded participants, blinded therapists, and intention-to-treat analysis. The risk associated with selective reporting was unclear, and none of the studies blinded the therapists or participants.

### 3.3. Water-Based Exercise versus Control

**Figure 2:**
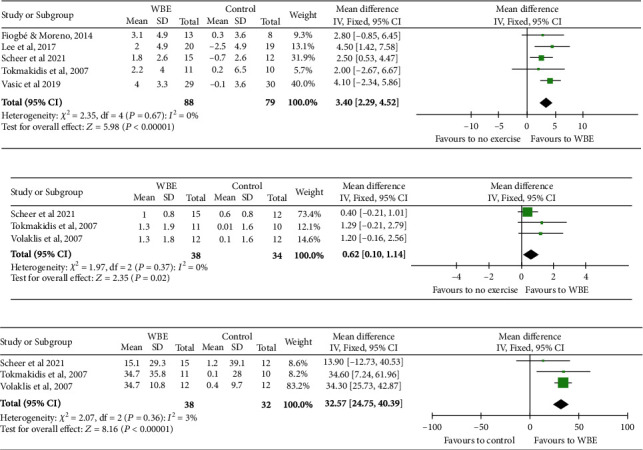
Forest plot showing the meta-analysis of WBE in comparison to no exercise group for (a) peak VO_2_, (b) exercise time, and (c) body strength.

Five studies assessed peak VO_2_ [14, 15, 28, 30, 32]. There were 88 patients in the water-based exercise group and 79 in the control group. The meta-analyses showed a significant improvement in peak VO_2_ of 3.4 mL/kg/min (95% CI, 2.3 to 4.5; *I*^2^ = 0%; 5 studies, *N* = 167; low-quality evidence, downgraded for risk of bias and imprecision, Figure 2(a) and *supplementary content*) for participants in the water-based exercise group versus the control group.

Three studies assessed exercise time [15, 30, 31]. There were 37 patients in the water-based exercise group and 32 in the control group. The meta-analyses showed a significant improvement in exercise time of 36 seconds (95% CI, 0.1 to 1.1; *I*^2^ = 0%; 3 studies, *N* = 69; low-quality evidence, downgraded for risk of bias and imprecision, Figure 2(b) and *supplementary content*) for participants in the water-based exercise group versus the control group.

Three studies assessed total body strength [15, 30, 31]. There were 37 patients in the water-based exercise group and 32 in the control group. The meta-analyses showed a significant improvement in total body strength of 32.2 kg (95% CI, 23.9 to 40.7; *I*^2^ = 3%; 3 studies, *N* = 69; low-quality evidence, downgraded for risk of bias and imprecision, Figure 2(c) and *supplementary content*) for participants in the water-based exercise group versus the control group.

### 3.4. Water-Based Exercise versus Land-Based Exercise

**Figure 3:**
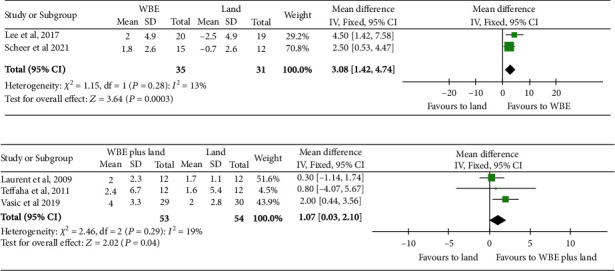
Forest plot showing the meta-analysis of water-based exercise alone in comparison to land-based exercise (a) and water-based exercise plus land-based exercises in comparison to land-based exercise (b) for peak VO_2_.

Two studies assessed peak VO_2_ as an outcome [15, 28]. There were 35 patients in the water-based exercise group and 31 in the land exercise group. The meta-analysis showed a significant difference in peak VO_2_ of 3.1 mL/kg/min (95% CI: 1.4 to 4.7, *N* = 66, *I*^2^ = 13%; 2 studies; low-quality evidence, downgraded for risk of bias and imprecision, *Supplementary content* for participants in the water-based exercise group compared with the land-based exercise group (Figure 3(a)).

### 3.5. Water-Based plus Land-Based Exercise versus Land-Based Exercise

Three studies assessed peak VO_2_ as an outcome [6, 14, 29]. There were 53 patients in the water-based plus land-based exercise group and 54 in the land-based exercise group. The meta-analysis showed a nonsignificant difference in peak VO_2_ of 1.1 mL/kg/min (95% CI: 0.03 to 2.1, *N* = 107, *I*^2^ = 19%; 3 studies; low-quality evidence, downgraded for risk of bias and imprecision, *Supplementary content*) for participants in the water-based plus land-based exercise group compared with the land exercise group (Figure 3(b)).

## 4. GRADE Assessments

The GRADE assessments are presented in the summary of findings table (*Supplementary content*). The quality of evidence for the outcome of the exercise capacity, measured by the peak VO_2_, exercise time, and total body strength, was determined to be low.

## 5. Discussion

The main results of our systematic review indicated that water-based exercise was efficient in improving peak VO_2_, exercise time, and muscle strength in patients with coronary artery disease when compared to nonexercising control. Additionally, water-based exercise was more effective than land-based exercise for peak VO_2_. Moreover, addition of water-based exercise to land-based exercise may be beneficial for further increasing the peak VO_2_ in patients with coronary artery disease when compared to land-based exercise alone.

Water-based exercise, for some time, was seen as potentially dangerous to patients with cardiovascular conditions. The main argument was the action of hydrostatic pressure and the consequent increase in venous return and cardiac overload. However, it is known that cardiac function improves during water immersion due to the increase in early diastolic filling and decrease in heart rate, resulting in improvements in stroke volume and ejection fraction [11]. Our results incite a positive discussion for water-based exercise as a potential intervention in cardiovascular rehabilitation. The magnitude of improvement in peak VO_2_ with water-based exercise (mean change: +2.55 mL/kg/min) was superior to the improvements in comparison to the no exercising group (mean change: -1.15 mL/kg/min). Furthermore, our analysis showed that improvements in peak VO_2_ with water-based exercise were superior to the improvements in comparison to land-based exercises (mean difference: 3.08 mL/kg/min). Thus, our systematic review with meta-analysis shows that water-based exercises could be a potential coadjuvant modality in the rehabilitation of patients with coronary artery disease. The eligibility of peak VO_2_ as our primary outcome is relevant because peak VO_2_ is a prognostic variable in patients with cardiovascular conditions [33, 34]. Improvements of 10% are associated with better prognosis in patients with cardiovascular conditions. Our meta-analysis showed a 20.1% of improvement in peak VO_2_ in the water-based exercise. In addition, the improvements generated by water-based exercise programs in exercise capacity can contribute to better performance in carrying out everyday activities [35].

Water-based exercise can help patients by providing a low-risk exercise environment that supports body weight and reduces the risk or fear of falling. In addition, it provides a playful environment with less joint overload and a lower risk for musculoskeletal injuries, stress relief, and confidence to perform activities. Water resistance increases muscle work when moving the body through water, and the warm water temperature, which may improve blood flow to muscles, may enable a higher intensity and duration of exercise, especially in people who have difficulty completing a land-based exercise training program [36]. Immersion promotes adjustments such as increased venous return, central venous pressure, and diastolic filling, as already mentioned, which may have promoted adaptations of the cardiorespiratory system favoring the improvement of functional capacity [32], in addition to peripheral adaptations of skeletal muscles [31]. Improvements in muscle strength were associated with reduced cardiovascular and all-cause mortality in people with coronary disease [2], suggesting that the muscle strength gains observed in our study have clinical value.

The results of this systematic review are in accordance with previous reviews that investigated the effect of water-based exercise on exercise capacity in chronic disease patients [12, 34] and physical functioning in older adults [37]. On the other hand, the results of this review differ from the results of Cugusi et al. [13], where no significant difference was detected for peak VO_2_, which is a prognostic variable in patients with cardiovascular conditions. In addition, in our study, we extended the previous review, including new studies and different analyses with participants of both sexes.

A pragmatic recommendation about water-based exercise in patients with coronary artery disease is not possible due to the low quality of the studies. Despite this, water-based exercise seems to be a potential tool in cardiac rehabilitation and deserves more investigation with new large-scale randomized controlled trials.

## 6. Limitations

It is important to address some limitations of our study. First, the included studies presented a small number of patients. Second, our quality assessment analysis showed a moderate risk of bias, and third, only 8,74% of the patients were women. Therefore, these results should not necessarily be generalized. Further high-quality randomized controlled trials are needed to better assess the effects of water-based exercise in coronary artery disease patients.

## 7. Conclusion

Considering the available data, our systematic review showed that water-based exercise alone was efficient to improve peak VO_2_, exercise time, and muscle strength in patients with coronary artery disease. Moreover, water-based exercise alone or plus land based-exercise was more effective than land-based exercise alone for peak VO_2_. Thus, water-based exercise seems to be a useful strategy to improve exercise capacity in coronary artery disease patients and may be viewed as an option to be included in rehabilitation programs. However, further well-controlled trials are needed to better understand the potential benefits of water-based exercise.

## Figures and Tables

**Table 1:** Study quality on the PEDro scale.

	Study	1^∗^	2	3	4	5	6	7	8	9	10	11	Total
1	Scheer et al., 2021 [15]	✓	✓	✓						✓	✓	✓	5
2	Vasić et al., 2019 [14]	✓	✓	✓					✓	✓	✓	✓	6
3	Lee et al., 2017 [28]		✓		✓					✓			3
4	Fiogbé and Moreno, 2014 [32]	✓		✓		✓		✓	✓		✓		5
5	Teffaha et al., 2011 [29]		✓		✓				✓		✓	✓	5
6	Laurent et al., 2009 [6]		✓		✓						✓	✓	4
7	Tokmakidis et al., 2008[30]		✓		✓				✓		✓	✓	5
8	Volaklis et al., 2007[31]		✓		✓				✓		✓	✓	5

1: eligibility criteria and source of participants; 2: random allocation; 3: concealed allocation; 4: baseline comparability; 5: blinded participants; 6: blinded therapists; 7: blind assessors; 8: adequate follow-up; 9: intention-to-treat analysis; 10: between-group comparisons; 11: point estimates and variability. ^∗^Item 1 does not contribute to the total score.

**Table 2:** Characteristics of the included studies.

	Study	Patients (*N* analysed, age, gender)	Muscle strength	Exercise tolerance	Key findings
1	Scheer et al., 2021 [15]	*N* = 45, 68 years, 80.7% male; 45 patients with stable CAD	Muscle strength (1-RM) Total body strength (biceps curl, latissimus dorsi pull-down, hamstring curl, and leg press)	Peak VO_2_, rating of perceived exertion, exercise duration	Both modes of exercise training improved exercise capacity (peak VO_2_) to a similar extent and the land group increased exercise time. Both the water and land groups increased leg strength, but only the land group significantly improved latissimus pull-down strength.
2	Vasić et al., 2019[14]	*N* = 89, 59.9 years, 77.5% male, 89 patients after a recent CAD event	—	Peak VO_2_	Both exercise modalities were associated with a significant improvement in peak VO_2_ as compared to controls.
3	Lee et al., 2017 [28]	*N* = 60, 73 years, 71.9% male; 60 patients with CAD	—	Peak VO_2_	Significant differences were observed in the change of cardiorespiratory fitness expressed as peak VO_2_ over 24 weeks among the groups. However, no significant differences in the change in these measures were found between the treadmill walking and aqua walking groups.
4	Fiogbé and Moreno, 2014 [32]	*N* = 26, 59.3 years, 100% male; 26 patients with stabilized CAD	—	Peak VO_2_	There was an increase in the values of peak VO_2_ and oxygen pulse.
5	Teffaha et al., 2011 [29]	*N* = 48, 52.45 years, 100% male; 24 patients with stabilized CAD and 24 patients with CHF	—	Peak VO_2_	Significant increases in peak VO_2_, heart rate, and power output were observed in all patients after rehabilitation in exercise test. The increase in left ventricular ejection fraction at rest, in heart rate, and power output at the exercise peak was slightly higher in the water group than in the land group.
6	Laurent et al., 2009 [6]	*N* = 48, 53.75 years, 100% male; 24 patients with stabilized CAD and 24 patients with CHF	—	Peak VO_2_	In every group, the cardiorespiratory capacity of patients was significantly increased after rehabilitation.
7	Tokmakidis et al., 2008 [30]	*N* = 21, 51.6 years, 100% male; 21 patients with CAD	Muscle strength (1-RM) Total body strength (pec-deck, seated row, lateral pull-down, chest press, leg extension, and leg flexion)	Peak VO_2_, 6 min water walking test, exercise duration	The exercise group improved their stress test time, peak VO_2_, and total body strength after the training period; detraining tended to reverse these positive adaptations. Resumption of training increased the beneficial effects obtained after the initial training period to exercise stress, peak VO_2_, and total strength. The patients in the control group did not show any significant alterations throughout the study.
8	Volaklis et al., 2007 [31]	*N* = 30, 54 years, 100% male; 30 patients with CAD and none participated in an exercise program at least 6 months before the study	Muscle strength (1-RM) Total body strength (bench press, pull-down, seated row, “peck-deck,” leg extension, and hamstring curl)	Exercise stress test on the treadmill, exercise duration	Water-based group improved exercise time and muscle strength in a similar manner compared to the patients who trained on land.

CAD: coronary artery disease; peak VO_2_: peak oxygen uptake; 1-RM: one repetition maximum.

**Table 3:** Characteristics of the intervention in the trials included in the review.

	Study	Modality	Intensity	Time/repetitions	Frequency (× per wk)	Length (wk)	Supervision
1	Scheer et al., 2021 [15]	Aquatic aerobic and strength exercises Xiphoid process 34.5°C TW	Aerobic exercise intensity commenced at 50 to 65% of the measured heart rate maximum in weeks 1 and 2 and increased to 60 to 65% in weeks 3 and 4, 60 to 70% in weeks 5 and 6, 70 to 80% in weeks 7 and 8, and 80% in weeks 9 to 12. Rating of perceived exertion was also used to guide exercise prescription and was progressed from 11 to 14 over the course of training. Resistance exercises were matched for muscle group between aquatic and land exercises, and the range of motion of the arm exercises for both groups was limited to the range allowed by the water level of the aquatic group. Resistance exercise ratings of perceived exertion targets were 12 to 15	60 min (5 min of light aerobic activity, 50 min of main program, and 5 min stretching)	3x	12	Yes

2	Vasić et al., 2019 [14]	Water-based endurance plus calisthenics exercise program Xiphoid process 32.8°C TW	Aerobic endurance and calisthenic exercises comprised at 60–80% peak heart rate	60 min (two 30 min sessions daily: 5 min of warm-up, 20 min of conditioning, and 5 min of cool down)	6x	2	Yes

3	Lee et al., 2017 [28]	Aqua walking Xiphoid process 30–32°C TW	15-17 bmp from the 50-65% of heart rate reserve and perceived exertion (11–14 in Borg's scale)	30 min	3x	24	Yes

4	Fiogbé and Moreno, 2014 [32]	Aquatic aerobic physical training Xiphoid process 30–33°C TW	80-110% of the first ventilatory threshold	30-50 min	3x	16	Yes

5	Teffaha et al., 2011 [29]	Endurance and callisthenic exercises 30–32°C TW	Target intensity heart rate recorded at the ventilatory threshold during the first exercise tolerance test	50 min	5x	3	Yes

6	Laurent et al., 2009 [6]	Aerobic exercises 30–32°C TW	60–70% of heart rate reserve	50 min	5x	3	Yes

7	Tokmakidis et al., 2008 [30]	Aerobic training and weight training 28-30°C TW	50–85% of maximal heart rate and 60–80% of rate of perceived exertion (11–14 in Borg's scale) to weight training	75 min (10 min of warm-up, 30-40 min of main program, 10 min of cool down)	4x	16	Yes

8	Volaklis et al., 2007 [31]	Aerobic training and resistance training 28-30°C TW	60–80% of maximal heart rate and 12 to 15 repetitions at 60% of 1-RM	60 min (10 min of warm-up, 5 min of stretching, 40 min of main program, 10 min of recreation, and 10 min of cool down)	4x	16	Yes

TW: temperature of water; bpm: beat per minute; 1-RM: one repetition maximum.

## Data Availability

No new data were generated or analyzed in support of this research.

## Supplementary Materials

Supplementary Materials (1) Grading of Recommendations Assessment, Development and Evaluation (GRADE). (2) Search strategy.
