# Supplementary material for: Water-Based Exercises on Peak Oxygen Consumption, Exercise Time, and Muscle Strength in Patients with Coronary Artery Disease: A Systematic Review with Meta-Analysis
Source: Cardiovasc Ther. 2023 Jun 26;2023:4305474. doi: 10.1155/2023/4305474 (PMC10317579; doi:10.1155/2023/4305474)
Supplement: Supplementary Materials — (1) Grading of Recommendations Assessment, Development and Evaluation (GRADE). (2) Search strategy. [file 4305474.f1.pdf]

Summary of findings:

WBE compared to Control for [CAD]

Patient or population: [CAD]

Setting:

Intervention: WBE

Comparison: Control

Outcomes	Anticipated absolute effects* (95% CI)		Relative effect (95% CI)	№ of participants (studies)	Certainty of the evidence (GRADE)	Comments
	Risk with Control	Risk with WBE				
Exercise time	The mean exercise time was **0**	MD **0.62 higher** (0.1 higher to 1.14 higher)	-	72 (3 RCTs)	⨁⨁◯◯ Low[a,b]	
Total strength	The mean total strength was **0**	MD **32.34 higher** (23.94 higher to 40.74 higher)	-	70 (3 RCTs)	⨁⨁◯◯ Low[a,b]	
VO2 Peak	The mean VO2 Peak was **0**	MD **3.4 higher** (2.29 higher to 4.52 higher)	-	167 (5 RCTs)	⨁⨁◯◯ Low[a,b]	

***The risk in the intervention group** (and its 95% confidence interval) is based on the assumed risk in the comparison group and the **relative effect** of the intervention (and its 95% CI).

CI: confidence interval; **MD:** mean difference

GRADE Working Group grades of evidence
High certainty: we are very confident that the true effect lies close to that of the estimate of the effect.
Moderate certainty: we are moderately confident in the effect estimate: the true effect is likely to be close to the estimate of the effect, but there is a possibility that it is substantially different.
Low certainty: our confidence in the effect estimate is limited: the true effect may be substantially different from the estimate of the effect.
Very low certainty: we have very little confidence in the effect estimate: the true effect is likely to be substantially different from the estimate of effect.

Explanations

a. Studies without allocation concealment, random allocation, and/or sample size calculation.

b. Total population size less than 400.

Summary of findings:

WBE + Land compared to Land for [CAD]

Patient or population: [CAD]

Setting:

Intervention: WBE + Land

Comparison: Land

Outcomes	Anticipated absolute effects* (95% CI)		Relative effect (95% CI)	№ of participants (studies)	Certainty of the evidence (GRADE)	Comments
	**Risk with Land**	**Risk with WBE + Land**				
VO2 Peak	The mean VO2 Peak was **0**	MD **1.07 higher** (0.03 higher to 2.1 higher)	-	107 (3 RCTs)	⨁⨁◯◯ Low[a,b]	

***The risk in the intervention group** (and its 95% confidence interval) is based on the assumed risk in the comparison group and the **relative effect** of the intervention (and its 95% CI).

CI: confidence interval; **MD:** mean difference

GRADE Working Group grades of evidence
High certainty: we are very confident that the true effect lies close to that of the estimate of the effect.
Moderate certainty: we are moderately confident in the effect estimate: the true effect is likely to be close to the estimate of the effect, but there is a possibility that it is substantially different.
Low certainty: our confidence in the effect estimate is limited: the true effect may be substantially different from the estimate of the effect.
Very low certainty: we have very little confidence in the effect estimate: the true effect is likely to be substantially different from the estimate of effect.

Explanations

a. Studies without allocation concealment, random allocation, and/or sample size calculation.

b. Total population size less than 400

Summary of findings:

WBE x Land compared to placebo for [CAD]

Patient or population: [CAD]

Setting:

Intervention: WBE x Land

Comparison: placebo

Outcomes	Anticipated absolute effects* (95% CI)		Relative effect (95% CI)	№ of participants (studies)	Certainty of the evidence (GRADE)	Comments
	**Risk with placebo**	**Risk with WBE x Land**				
VO2 Peak	The mean VO2 Peak was **0**	MD **3.08 higher** (1.42 higher to 4.74 higher)	-	66 (2 RCTs)	⨁⨁◯◯ Low[a,b]	

***The risk in the intervention group** (and its 95% confidence interval) is based on the assumed risk in the comparison group and the **relative effect** of the intervention (and its 95% CI).

CI: confidence interval; **MD:** mean difference

GRADE Working Group grades of evidence
High certainty: we are very confident that the true effect lies close to that of the estimate of the effect.
Moderate certainty: we are moderately confident in the effect estimate: the true effect is likely to be close to the estimate of the effect, but there is a possibility that it is substantially different.
Low certainty: our confidence in the effect estimate is limited: the true effect may be substantially different from the estimate of the effect.
Very low certainty: we have very little confidence in the effect estimate: the true effect is likely to be substantially different from the estimate of effect.

Explanations

a. Studies without allocation concealment, random allocation, and/or sample size calculation.

b. Total population size less than 400.

SUPPLEMENTARY CONTENT

Search strategy PubMed

(("Coronary Artery Disease"[MeSH Terms] OR "Myocardial Ischemia"[MeSH Terms] OR "Myocardial Infarction"[MeSH Terms] OR ("Coronary Artery Disease"[MeSH Terms] OR ("coronary"[All Fields] AND "artery"[All Fields] AND "disease"[All Fields]) OR "Coronary Artery Disease"[All Fields] OR ("artery"[All Fields] AND "disease"[All Fields] AND "coronary"[All Fields]) OR "artery disease coronary"[All Fields] OR ("Coronary Artery Disease"[MeSH Terms] OR ("coronary"[All Fields] AND "artery"[All Fields] AND "disease"[All Fields]) OR "Coronary Artery Disease"[All Fields] OR ("artery"[All Fields] AND "diseases"[All Fields] AND "coronary"[All Fields]) OR "artery diseases coronary"[All Fields]) OR ("Coronary Artery Disease"[MeSH Terms] OR ("coronary"[All Fields] AND "artery"[All Fields] AND "disease"[All Fields]) OR "Coronary Artery Disease"[All Fields] OR ("coronary"[All Fields] AND "artery"[All Fields] AND "diseases"[All Fields]) OR "coronary artery diseases"[All Fields]) OR ("Coronary Artery Disease"[MeSH Terms] OR ("coronary"[All Fields] AND "artery"[All Fields] AND "disease"[All Fields]) OR "Coronary Artery Disease"[All Fields] OR ("left"[All Fields] AND "main"[All Fields] AND "coronary"[All Fields] AND "artery"[All Fields] AND "disease"[All Fields]) OR "left main coronary artery disease"[All Fields]) OR ("Coronary Artery Disease"[MeSH Terms] OR ("coronary"[All Fields] AND "artery"[All Fields] AND "disease"[All Fields]) OR "Coronary Artery Disease"[All Fields] OR ("left"[All Fields] AND "main"[All Fields] AND "disease"[All Fields]) OR "left main disease"[All Fields]) OR ("Coronary Artery Disease"[MeSH Terms] OR ("coronary"[All Fields] AND "artery"[All Fields] AND "disease"[All Fields]) OR "Coronary Artery Disease"[All Fields] OR ("left"[All Fields] AND "main"[All Fields] AND "diseases"[All Fields]) OR "left main diseases"[All Fields]) OR ("Coronary Artery Disease"[MeSH Terms] OR ("coronary"[All Fields] AND "artery"[All Fields] AND "disease"[All Fields]) OR "Coronary Artery Disease"[All Fields] OR ("left"[All Fields] AND "main"[All Fields] AND "coronary"[All Fields] AND "disease"[All Fields]) OR "left main coronary disease"[All Fields]) OR ("Coronary Artery Disease"[MeSH Terms] OR ("coronary"[All Fields] AND "artery"[All Fields] AND "disease"[All Fields]) OR "Coronary Artery Disease"[All Fields] OR ("coronary"[All Fields] AND "arteriosclerosis"[All Fields]) OR "coronary arteriosclerosis"[All Fields]) OR ("Coronary Artery Disease"[MeSH Terms] OR ("coronary"[All Fields] AND "artery"[All Fields] AND "disease"[All Fields]) OR "Coronary Artery Disease"[All Fields] OR ("arterioscleroses"[All Fields] AND "coronary"[All Fields])) OR ("Coronary Artery Disease"[MeSH Terms] OR ("coronary"[All Fields] AND "artery"[All Fields] AND "disease"[All Fields]) OR "Coronary Artery Disease"[All Fields] OR ("coronary"[All Fields] AND "arterioscleroses"[All Fields])) OR ("Coronary Artery Disease"[MeSH Terms] OR ("coronary"[All Fields] AND "artery"[All Fields] AND "disease"[All Fields]) OR "Coronary Artery Disease"[All Fields] OR ("atherosclerosis"[All Fields] AND "coronary"[All Fields]) OR "atherosclerosis coronary"[All Fields]) OR ("Coronary Artery Disease"[MeSH Terms] OR ("coronary"[All Fields] AND "artery"[All Fields] AND "disease"[All Fields]) OR "Coronary Artery Disease"[All Fields] OR ("atheroscleroses"[All Fields] AND "coronary"[All Fields])) OR ("Coronary Artery Disease"[MeSH Terms] OR ("coronary"[All Fields] AND "artery"[All Fields] AND "disease"[All Fields]) OR "Coronary Artery Disease"[All Fields] OR ("coronary"[All Fields] AND "atheroscleroses"[All Fields])) OR ("Coronary Artery Disease"[MeSH Terms] OR ("coronary"[All Fields] AND "artery"[All Fields] AND "disease"[All Fields]) OR "Coronary Artery Disease"[All Fields] OR ("coronary"[All Fields] AND "atherosclerosis"[All Fields]) OR "coronary atherosclerosis"[All Fields]) OR

("Coronary Artery Disease"[MeSH Terms] OR ("coronary"[All Fields] AND "artery"[All Fields] AND "disease"[All Fields]) OR "Coronary Artery Disease"[All Fields] OR ("arteriosclerosis"[All Fields] AND "coronary"[All Fields]) OR "arteriosclerosis coronary"[All Fields]) OR ("Myocardial Ischemia"[MeSH Terms] OR ("myocardial"[All Fields] AND "ischemia"[All Fields]) OR "Myocardial Ischemia"[All Fields] OR ("ischemia"[All Fields] AND "myocardial"[All Fields]) OR "ischemia myocardial"[All Fields]) OR ("Myocardial Ischemia"[MeSH Terms] OR ("myocardial"[All Fields] AND "ischemia"[All Fields]) OR "Myocardial Ischemia"[All Fields] OR ("ischemias"[All Fields] AND "myocardial"[All Fields]) OR "ischemias myocardial"[All Fields]) OR ("Myocardial Ischemia"[MeSH Terms] OR ("myocardial"[All Fields] AND "ischemia"[All Fields]) OR "Myocardial Ischemia"[All Fields] OR ("myocardial"[All Fields] AND "ischemias"[All Fields]) OR "myocardial ischemias"[All Fields]) OR ("ischaemic heart disease"[All Fields] OR "Myocardial Ischemia"[MeSH Terms] OR ("myocardial"[All Fields] AND "ischemia"[All Fields]) OR "Myocardial Ischemia"[All Fields] OR ("ischemic"[All Fields] AND "heart"[All Fields] AND "disease"[All Fields]) OR "ischemic heart disease"[All Fields] OR "Coronary Artery Disease"[MeSH Terms] OR ("coronary"[All Fields] AND "artery"[All Fields] AND "disease"[All Fields]) OR "Coronary Artery Disease"[All Fields] OR ("ischemic"[All Fields] AND "heart"[All Fields] AND "disease"[All Fields])) OR ("Myocardial Ischemia"[MeSH Terms] OR ("myocardial"[All Fields] AND "ischemia"[All Fields]) OR "Myocardial Ischemia"[All Fields] OR ("heart"[All Fields] AND "disease"[All Fields] AND "ischemic"[All Fields]) OR "heart disease ischemic"[All Fields]) OR ("Myocardial Ischemia"[MeSH Terms] OR ("myocardial"[All Fields] AND "ischemia"[All Fields]) OR "Myocardial Ischemia"[All Fields] OR ("disease"[All Fields] AND "ischemic"[All Fields] AND "heart"[All Fields]) OR "disease ischemic heart"[All Fields]) OR ("Myocardial Ischemia"[MeSH Terms] OR ("myocardial"[All Fields] AND "ischemia"[All Fields]) OR "Myocardial Ischemia"[All Fields] OR ("diseases"[All Fields] AND "ischemic"[All Fields] AND "heart"[All Fields]) OR "diseases ischemic heart"[All Fields]) OR ("Myocardial Ischemia"[MeSH Terms] OR ("myocardial"[All Fields] AND "ischemia"[All Fields]) OR "Myocardial Ischemia"[All Fields] OR ("heart"[All Fields] AND "diseases"[All Fields] AND "ischemic"[All Fields]) OR "heart diseases ischemic"[All Fields]) OR ("ischaemic heart diseases"[All Fields] OR "Myocardial Ischemia"[MeSH Terms] OR ("myocardial"[All Fields] AND "ischemia"[All Fields]) OR "Myocardial Ischemia"[All Fields] OR ("ischemic"[All Fields] AND "heart"[All Fields] AND "diseases"[All Fields]) OR "ischemic heart diseases"[All Fields]) OR ("Myocardial Infarction"[MeSH Terms] OR ("myocardial"[All Fields] AND "infarction"[All Fields]) OR "Myocardial Infarction"[All Fields] OR ("infarction"[All Fields] AND "myocardial"[All Fields]) OR "infarction myocardial"[All Fields]) OR ("Myocardial Infarction"[MeSH Terms] OR ("myocardial"[All Fields] AND "infarction"[All Fields]) OR "Myocardial Infarction"[All Fields] OR ("infarctions"[All Fields] AND "myocardial"[All Fields]) OR "infarctions myocardial"[All Fields]) OR ("Myocardial Infarction"[MeSH Terms] OR ("myocardial"[All Fields] AND "infarction"[All Fields]) OR "Myocardial Infarction"[All Fields] OR ("myocardial"[All Fields] AND "infarctions"[All Fields]) OR "myocardial infarctions"[All Fields]) OR ("Myocardial Infarction"[MeSH Terms] OR ("myocardial"[All Fields] AND "infarction"[All Fields]) OR "Myocardial Infarction"[All Fields] OR ("cardiovascular"[All Fields] AND "stroke"[All Fields]) OR "cardiovascular stroke"[All Fields]) OR ("Myocardial Infarction"[MeSH Terms] OR ("myocardial"[All Fields] AND "infarction"[All Fields]) OR "Myocardial Infarction"[All Fields] OR ("cardiovascular"[All Fields] AND "strokes"[All Fields])) OR ("Myocardial Infarction"[MeSH Terms] OR ("myocardial"[All Fields] AND "infarction"[All Fields]) OR "Myocardial Infarction"[All Fields] OR ("stroke"[All Fields] AND "cardiovascular"[All Fields]) OR "stroke cardiovascular"[All Fields]) OR ("Myocardial Infarction"[MeSH Terms] OR ("myocardial"[All Fields] AND "infarction"[All Fields]) OR

"Myocardial Infarction"[All Fields] OR ("strokes"[All Fields] AND "cardiovascular"[All Fields]) OR "strokes cardiovascular"[All Fields]) OR ("Myocardial Infarction"[MeSH Terms] OR ("myocardial"[All Fields] AND "infarction"[All Fields]) OR "Myocardial Infarction"[All Fields] OR ("myocardial"[All Fields] AND "infarct"[All Fields]) OR "myocardial infarct"[All Fields]) OR ("Myocardial Infarction"[MeSH Terms] OR ("myocardial"[All Fields] AND "infarction"[All Fields]) OR "Myocardial Infarction"[All Fields] OR ("infarct"[All Fields] AND "myocardial"[All Fields]) OR "infarct myocardial"[All Fields]) OR ("Myocardial Infarction"[MeSH Terms] OR ("myocardial"[All Fields] AND "infarction"[All Fields]) OR "Myocardial Infarction"[All Fields] OR ("infarcts"[All Fields] AND "myocardial"[All Fields]) OR "infarcts myocardial"[All Fields]) OR ("Myocardial Infarction"[MeSH Terms] OR ("myocardial"[All Fields] AND "infarction"[All Fields]) OR "Myocardial Infarction"[All Fields] OR ("myocardial"[All Fields] AND "infarcts"[All Fields]) OR "myocardial infarcts"[All Fields]) OR ("Myocardial Infarction"[MeSH Terms] OR ("myocardial"[All Fields] AND "infarction"[All Fields]) OR "Myocardial Infarction"[All Fields] OR ("heart"[All Fields] AND "attack"[All Fields]) OR "heart attack"[All Fields]) OR ("Myocardial Infarction"[MeSH Terms] OR ("myocardial"[All Fields] AND "infarction"[All Fields]) OR "Myocardial Infarction"[All Fields] OR ("heart"[All Fields] AND "attacks"[All Fields]) OR "heart attacks"[All Fields]))) AND ("aquatic"[All Fields] OR "aquatically"[All Fields] OR "aquatics"[All Fields] OR ("aquatic therapy"[MeSH Terms] OR ("aquatic"[All Fields] AND "therapy"[All Fields]) OR "aquatic therapy"[All Fields]) OR (("aquatic"[All Fields] OR "aquatically"[All Fields] OR "aquatics"[All Fields]) AND ("exercise"[MeSH Terms] OR "exercise"[All Fields] OR "exercises"[All Fields] OR "exercise therapy"[MeSH Terms] OR ("exercise"[All Fields] AND "therapy"[All Fields]) OR "exercise therapy"[All Fields] OR "exercise s"[All Fields] OR "exercised"[All Fields] OR "exerciser"[All Fields] OR "exercisers"[All Fields] OR "exercising"[All Fields])) OR (("aquatic"[All Fields] OR "aquatically"[All Fields] OR "aquatics"[All Fields]) AND ("physical therapy modalities"[MeSH Terms] OR ("physical"[All Fields] AND "therapy"[All Fields] AND "modalities"[All Fields]) OR "physical therapy modalities"[All Fields] OR "physiotherapies"[All Fields] OR "physiotherapy"[All Fields])) OR ("water-based"[All Fields] AND ("exercise"[MeSH Terms] OR "exercise"[All Fields] OR "exercises"[All Fields] OR "exercise therapy"[MeSH Terms] OR ("exercise"[All Fields] AND "therapy"[All Fields]) OR "exercise therapy"[All Fields] OR "exercise s"[All Fields] OR "exercised"[All Fields] OR "exerciser"[All Fields] OR "exercisers"[All Fields] OR "exercising"[All Fields])) OR ("hydrotherapies"[All Fields] OR "Hydrotherapy"[MeSH Terms] OR "Hydrotherapy"[All Fields] OR ("Hydrotherapy"[MeSH Terms] OR "Hydrotherapy"[All Fields] OR ("whirlpool"[All Fields] AND "baths"[All Fields]) OR "whirlpool baths"[All Fields]) OR ("Hydrotherapy"[MeSH Terms] OR "Hydrotherapy"[All Fields] OR ("bath"[All Fields] AND "whirlpool"[All Fields]) OR "bath whirlpool"[All Fields]) OR ("Hydrotherapy"[MeSH Terms] OR "Hydrotherapy"[All Fields] OR ("baths"[All Fields] AND "whirlpool"[All Fields]) OR "baths whirlpool"[All Fields]) OR ("Hydrotherapy"[MeSH Terms] OR "Hydrotherapy"[All Fields] OR ("whirlpool"[All Fields] AND "bath"[All Fields]) OR "whirlpool bath"[All Fields])) OR "Hydrotherapy"[MeSH Terms])) AND ("Coronary Artery Disease"[MeSH Terms] OR "Myocardial Ischemia"[MeSH Terms] OR "Myocardial Infarction"[MeSH Terms] OR ("Coronary Artery Disease"[MeSH Terms] OR ("coronary"[All Fields] AND "artery"[All Fields] AND "disease"[All Fields]) OR "Coronary Artery Disease"[All Fields] OR ("artery"[All Fields] AND "disease"[All Fields] AND "coronary"[All Fields]) OR "artery disease coronary"[All Fields] OR ("Coronary Artery Disease"[MeSH Terms] OR ("coronary"[All Fields] AND "artery"[All Fields] AND "disease"[All Fields]) OR "Coronary Artery Disease"[All Fields] OR ("artery"[All Fields] AND "diseases"[All Fields] AND "coronary"[All Fields]) OR "artery diseases coronary"[All Fields]) OR ("Coronary Artery Disease"[MeSH Terms] OR ("coronary"[All Fields] AND

"artery"[All Fields] AND "disease"[All Fields]) OR "Coronary Artery Disease"[All Fields] OR ("coronary"[All Fields] AND "artery"[All Fields] AND "diseases"[All Fields]) OR "coronary artery diseases"[All Fields]) OR ("Coronary Artery Disease"[MeSH Terms] OR ("coronary"[All Fields] AND "artery"[All Fields] AND "disease"[All Fields]) OR "Coronary Artery Disease"[All Fields] OR ("left"[All Fields] AND "main"[All Fields] AND "coronary"[All Fields] AND "artery"[All Fields] AND "disease"[All Fields]) OR "left main coronary artery disease"[All Fields]) OR ("Coronary Artery Disease"[MeSH Terms] OR ("coronary"[All Fields] AND "artery"[All Fields] AND "disease"[All Fields]) OR "Coronary Artery Disease"[All Fields] OR ("left"[All Fields] AND "main"[All Fields] AND "disease"[All Fields]) OR "left main disease"[All Fields]) OR ("Coronary Artery Disease"[MeSH Terms] OR ("coronary"[All Fields] AND "artery"[All Fields] AND "disease"[All Fields]) OR "Coronary Artery Disease"[All Fields] OR ("left"[All Fields] AND "main"[All Fields] AND "diseases"[All Fields]) OR "left main diseases"[All Fields]) OR ("Coronary Artery Disease"[MeSH Terms] OR ("coronary"[All Fields] AND "artery"[All Fields] AND "disease"[All Fields]) OR "Coronary Artery Disease"[All Fields] OR ("left"[All Fields] AND "main"[All Fields] AND "coronary"[All Fields] AND "disease"[All Fields]) OR "left main coronary disease"[All Fields]) OR ("Coronary Artery Disease"[MeSH Terms] OR ("coronary"[All Fields] AND "artery"[All Fields] AND "disease"[All Fields]) OR "Coronary Artery Disease"[All Fields] OR ("coronary"[All Fields] AND "arteriosclerosis"[All Fields]) OR "coronary arteriosclerosis"[All Fields]) OR ("Coronary Artery Disease"[MeSH Terms] OR ("coronary"[All Fields] AND "artery"[All Fields] AND "disease"[All Fields]) OR "Coronary Artery Disease"[All Fields] OR ("arterioscleroses"[All Fields] AND "coronary"[All Fields])) OR ("Coronary Artery Disease"[MeSH Terms] OR ("coronary"[All Fields] AND "artery"[All Fields] AND "disease"[All Fields]) OR "Coronary Artery Disease"[All Fields] OR ("coronary"[All Fields] AND "arterioscleroses"[All Fields])) OR ("Coronary Artery Disease"[MeSH Terms] OR ("coronary"[All Fields] AND "artery"[All Fields] AND "disease"[All Fields]) OR "Coronary Artery Disease"[All Fields] OR ("atherosclerosis"[All Fields] AND "coronary"[All Fields]) OR "atherosclerosis coronary"[All Fields]) OR ("Coronary Artery Disease"[MeSH Terms] OR ("coronary"[All Fields] AND "artery"[All Fields] AND "disease"[All Fields]) OR "Coronary Artery Disease"[All Fields] OR ("atheroscleroses"[All Fields] AND "coronary"[All Fields])) OR ("Coronary Artery Disease"[MeSH Terms] OR ("coronary"[All Fields] AND "artery"[All Fields] AND "disease"[All Fields]) OR "Coronary Artery Disease"[All Fields] OR ("coronary"[All Fields] AND "atheroscleroses"[All Fields])) OR ("Coronary Artery Disease"[MeSH Terms] OR ("coronary"[All Fields] AND "artery"[All Fields] AND "disease"[All Fields]) OR "Coronary Artery Disease"[All Fields] OR ("coronary"[All Fields] AND "atherosclerosis"[All Fields]) OR "coronary atherosclerosis"[All Fields]) OR ("Coronary Artery Disease"[MeSH Terms] OR ("coronary"[All Fields] AND "artery"[All Fields] AND "disease"[All Fields]) OR "Coronary Artery Disease"[All Fields] OR ("arteriosclerosis"[All Fields] AND "coronary"[All Fields]) OR "arteriosclerosis coronary"[All Fields]) OR ("Myocardial Ischemia"[MeSH Terms] OR ("myocardial"[All Fields] AND "ischemia"[All Fields]) OR "Myocardial Ischemia"[All Fields] OR ("ischemia"[All Fields] AND "myocardial"[All Fields]) OR "ischemia myocardial"[All Fields]) OR ("Myocardial Ischemia"[MeSH Terms] OR ("myocardial"[All Fields] AND "ischemia"[All Fields]) OR "Myocardial Ischemia"[All Fields] OR ("ischemias"[All Fields] AND "myocardial"[All Fields]) OR "ischemias myocardial"[All Fields]) OR ("Myocardial Ischemia"[MeSH Terms] OR ("myocardial"[All Fields] AND "ischemia"[All Fields]) OR "Myocardial Ischemia"[All Fields] OR ("myocardial"[All Fields] AND "ischemias"[All Fields]) OR "myocardial ischemias"[All Fields]) OR ("ischaemic heart disease"[All Fields] OR "Myocardial Ischemia"[MeSH Terms] OR ("myocardial"[All Fields] AND "ischemia"[All Fields]) OR "Myocardial Ischemia"[All Fields] OR ("ischemic"[All Fields]

AND "heart"[All Fields] AND "disease"[All Fields]) OR "ischemic heart disease"[All Fields] OR "Coronary Artery Disease"[MeSH Terms] OR ("coronary"[All Fields] AND "artery"[All Fields] AND "disease"[All Fields]) OR "Coronary Artery Disease"[All Fields] OR ("ischemic"[All Fields] AND "heart"[All Fields] AND "disease"[All Fields])) OR ("Myocardial Ischemia"[MeSH Terms] OR ("myocardial"[All Fields] AND "ischemia"[All Fields]) OR "Myocardial Ischemia"[All Fields] OR ("heart"[All Fields] AND "disease"[All Fields] AND "ischemic"[All Fields]) OR "heart disease ischemic"[All Fields]) OR ("Myocardial Ischemia"[MeSH Terms] OR ("myocardial"[All Fields] AND "ischemia"[All Fields]) OR "Myocardial Ischemia"[All Fields] OR ("disease"[All Fields] AND "ischemic"[All Fields] AND "heart"[All Fields]) OR "disease ischemic heart"[All Fields]) OR ("Myocardial Ischemia"[MeSH Terms] OR ("myocardial"[All Fields] AND "ischemia"[All Fields]) OR "Myocardial Ischemia"[All Fields] OR ("diseases"[All Fields] AND "ischemic"[All Fields] AND "heart"[All Fields]) OR "diseases ischemic heart"[All Fields]) OR ("Myocardial Ischemia"[MeSH Terms] OR ("myocardial"[All Fields] AND "ischemia"[All Fields]) OR "Myocardial Ischemia"[All Fields] OR ("heart"[All Fields] AND "diseases"[All Fields] AND "ischemic"[All Fields]) OR "heart diseases ischemic"[All Fields]) OR ("ischaemic heart diseases"[All Fields] OR "Myocardial Ischemia"[MeSH Terms] OR ("myocardial"[All Fields] AND "ischemia"[All Fields]) OR "Myocardial Ischemia"[All Fields] OR ("ischemic"[All Fields] AND "heart"[All Fields] AND "diseases"[All Fields]) OR "ischemic heart diseases"[All Fields]) OR ("Myocardial Infarction"[MeSH Terms] OR ("myocardial"[All Fields] AND "infarction"[All Fields]) OR "Myocardial Infarction"[All Fields] OR ("infarction"[All Fields] AND "myocardial"[All Fields]) OR "infarction myocardial"[All Fields]) OR ("Myocardial Infarction"[MeSH Terms] OR ("myocardial"[All Fields] AND "infarction"[All Fields]) OR "Myocardial Infarction"[All Fields] OR ("infarctions"[All Fields] AND "myocardial"[All Fields]) OR "infarctions myocardial"[All Fields]) OR ("Myocardial Infarction"[MeSH Terms] OR ("myocardial"[All Fields] AND "infarction"[All Fields]) OR "Myocardial Infarction"[All Fields] OR ("myocardial"[All Fields] AND "infarctions"[All Fields]) OR "myocardial infarctions"[All Fields]) OR ("Myocardial Infarction"[MeSH Terms] OR ("myocardial"[All Fields] AND "infarction"[All Fields]) OR "Myocardial Infarction"[All Fields] OR ("cardiovascular"[All Fields] AND "stroke"[All Fields]) OR "cardiovascular stroke"[All Fields]) OR ("Myocardial Infarction"[MeSH Terms] OR ("myocardial"[All Fields] AND "infarction"[All Fields]) OR "Myocardial Infarction"[All Fields] OR ("cardiovascular"[All Fields] AND "strokes"[All Fields])) OR ("Myocardial Infarction"[MeSH Terms] OR ("myocardial"[All Fields] AND "infarction"[All Fields]) OR "Myocardial Infarction"[All Fields] OR ("stroke"[All Fields] AND "cardiovascular"[All Fields]) OR "stroke cardiovascular"[All Fields]) OR ("Myocardial Infarction"[MeSH Terms] OR ("myocardial"[All Fields] AND "infarction"[All Fields]) OR "Myocardial Infarction"[All Fields] OR ("strokes"[All Fields] AND "cardiovascular"[All Fields]) OR "strokes cardiovascular"[All Fields]) OR ("Myocardial Infarction"[MeSH Terms] OR ("myocardial"[All Fields] AND "infarction"[All Fields]) OR "Myocardial Infarction"[All Fields] OR ("myocardial"[All Fields] AND "infarct"[All Fields]) OR "myocardial infarct"[All Fields]) OR ("Myocardial Infarction"[MeSH Terms] OR ("myocardial"[All Fields] AND "infarction"[All Fields]) OR "Myocardial Infarction"[All Fields] OR ("infarct"[All Fields] AND "myocardial"[All Fields]) OR "infarct myocardial"[All Fields]) OR ("Myocardial Infarction"[MeSH Terms] OR ("myocardial"[All Fields] AND "infarction"[All Fields]) OR "Myocardial Infarction"[All Fields] OR ("infarcts"[All Fields] AND "myocardial"[All Fields]) OR "infarcts myocardial"[All Fields]) OR ("Myocardial Infarction"[MeSH Terms] OR ("myocardial"[All Fields] AND "infarction"[All Fields]) OR "Myocardial Infarction"[All Fields] OR ("myocardial"[All Fields] AND "infarcts"[All Fields]) OR "myocardial infarcts"[All Fields]) OR ("Myocardial Infarction"[MeSH Terms] OR ("myocardial"[All Fields] AND "infarction"[All Fields]) OR "Myocardial Infarction"[All

Fields] OR ("heart"[All Fields] AND "attack"[All Fields]) OR "heart attack"[All Fields]) OR ("Myocardial Infarction"[MeSH Terms] OR ("myocardial"[All Fields] AND "infarction"[All Fields]) OR "Myocardial Infarction"[All Fields] OR ("heart"[All Fields] AND "attacks"[All Fields]) OR "heart attacks"[All Fields])))

Search strategy EMBASE

('ischemic heart disease'/exp OR 'coronary artery insufficiency' OR 'coronary artery occlusive disease' OR 'coronary heart disease' OR 'coronary insufficiency' OR 'coronary occlusive disease' OR 'heart disease, coronary' OR 'heart disease, ischaemic' OR 'heart disease, ischemic' OR 'ischaemia heart disease' OR 'ischaemic cardiac disease' OR 'ischaemic cardial disease' OR 'ischaemic cardiopathy' OR 'ischaemic heart disease' OR 'ischemia heart disease' OR 'ischemic cardiac disease' OR 'ischemic cardial disease' OR 'ischemic cardiopathy' OR 'ischemic heart disease') AND ('hydrotherapy'/exp OR 'hydrotherapy' OR 'water immersion therapy' OR 'aquatic therapy'/exp OR 'aquatic exercise therapy' OR 'aquatic therapy' OR 'pool exercise therapy' OR 'pool therapy' OR 'water exercise therapy' OR 'water based exercise'/exp) AND ('randomized controlled trial'/exp OR 'controlled trial, randomized' OR 'randomised controlled study' OR 'randomised controlled trial' OR 'randomized controlled study' OR 'randomized controlled trial' OR 'trial, randomized controlled')
